# How Successful Is Surgical Sperm Retrieval in Klinefelter Syndrome?

**DOI:** 10.3389/frph.2021.636629

**Published:** 2021-02-18

**Authors:** Yamini Kailash, Amr Abdel Raheem, Sheryl T. Homa

**Affiliations:** ^1^Department of Urology, Guy's & St. Thomas' NHS Foundation Trust, London, United Kingdom; ^2^Andrology Department, Cairo University Hospital, Cairo, Egypt; ^3^Institute of Women's Health, University College London, London, United Kingdom; ^4^Faculty of Sciences, School of Biosciences, University of Kent, Canterbury, United Kingdom; ^5^Andrology Solutions, London, United Kingdom

**Keywords:** Klinefelter syndrome, surgical sperm retrieval, testicular sperm extraction, TESE, MicroTESE, hormone stimulation

## Abstract

Klinefelter Syndrome (KS) is characterized by the presence of an extra X chromosome. It was first diagnosed in 1942 in a group of azoospermic men. KS is the most common chromosomal abnormality encountered in infertile men and accounts for more than 10% of the causes of azoospermia. Men who are azoospermic may still father children via testicular sperm extraction followed by intracytoplasmic sperm injection (ICSI). This review article summarizes the success rates of the available techniques for surgical sperm retrieval (SSR) in KS including conventional testicular sperm extraction (cTESE) and micro testicular sperm extraction (mTESE), as well as the risks of these procedures for future fertility. The evidence indicates that the SSR rate is as successful in non-mosaic men with KS as those with normal karyotypes, with retrieval rates of up to 55% reported. The influence of different factors that affect the chances of a successful outcome are discussed. In particular, the impact of aneuploidy rate, physical characteristics, co-morbidities, reproductive endocrine balance and the use of different hormone management therapies are highlighted. Evidence is presented to suggest that the single most significant determinant for successful SSR is the age of the patient. The success of SSR is also influenced by surgical technique and operative time, as well as the skills of the surgeon and embryology team. Rescue mTESE may be used successfully following failed TESE in KS patients in combination with hormone stimulation.

## Introduction

In 1942, Harry Klinefelter published a case series of 9 syndromic men with gynaecomastia, hypogonadism and infertility (1). This was subsequently shown to be associated with sex chromosome anomalies (2). The term “Klinefelter syndrome” (KS) is used to describe a group of chromosomal disorders which is characterized by at least one extra X chromosome as opposed to the normal male karyotype of 46, XY. KS is the most common chromosomal abnormality in men, with a prevalence ranging from 1 in 500 to 1 in 700 (3) and is diagnosed in approximately 3-4% of infertile men and more than 10% of azoospermic men (4). KS is usually acquired through non-disjunction during parental gametogenesis of either paternal (53%) or maternal (44%) origin (5). KS men typically have hypergonadotropic hypogonadism and atrophic testes with progressive degeneration of testicular function. Testicular biopsy shows tubular hyalinisation with thickening of the basement membrane, Leydig cell hyperplasia and a Sertoli cell only phenotype (6). Biopsies of 47, XXY fetal testes show comparatively reduced numbers of germ cells. At puberty there is an acceleration in the deterioration of both germ cell lines apparently due to activation of the hypothalamic-pituitary-gonadal (HPG) axis and stimulation of gonadal tissue (7).

Gene cluster and pathway analysis showed four possible mechanisms responsible for reduced spermatogenesis in KS patients: impaired development of spermatogonia to mature spermatozoa, defects in the testis architecture, pathophysiology of the testis environment, and apoptosis of the germinal and somatic cells (7). Men with KS are likely to be infertile due to impaired spermatogenesis and are usually diagnosed with non-obstructive azoospermia (NOA) (8). For some time it has been established that localized foci of spermatogenesis exist within the testes in KS men (7, 9), however the origins of this process remain to be established. It has been proposed that these foci arise from 47XXY spermatogonia as there is some evidence demonstrating these cells are capable of completing meiosis (10) although this has been disputed by others (11, 12). If this were the case, 50% of resulting sperm would be expected to be aneuploid, with the remaining 50% containing a single sex chromosome. An alternative hypothesis supported by both mouse (13) and human (12) studies, suggests spermatogenic foci can only arise from clones of euploid spermatogonia (47XY) that have lost the surplus X chromosome either as a random event or during misalignment on the spindle microtubules during mitosis, potentially resulting in euploid spermatozoa. The evidence tends to favor the second theory, since there is no data to suggest a significant increase in aneuploidy rate or alteration in the sex ratio of the children born from KS fathers, which would be expected from offspring derived from 47XXY spermatogonia (14, 15).

Current practice for management of infertility for men with azoospermia generally includes TESE in order to retrieve sperm where there may be localized foci of spermatognesis. Because of the pathology associated with KS, TESE has resulted in limited success, however the implementation of the more advanced technique of micro TESE has increased recovery rates in these men. This review aims to discuss the success rates of the different types of SSR methods and to discuss the factors that may influence the outcome.

## Success Rate of SSR in Ks, Optimisation and Rescue TESE

Adult men with KS who are azoospermic may have small, patchy islands of spermatogenesis in the testes (7). SSR aims to target these areas to harvest spermatozoa for use with assisted conception treatment. The process of cTESE involves making a median raphe incision, delivery of both testes and taking multiple random testicular biopsies after making multiple small 1 cm incisions in the tunica albuginea. The tissue is then processed to milk any spermatozoa from the seminiferous tubules. It should be emphasized that while SSR is generally safe, complications may include infection, haematoma, chronic pain, testicular atrophy, and hypogonadism requiring hormone replacement (16, 17). Testicular atrophy may occur due to removal of too much of the testicular parenchyma and compromisation of the testicular blood supply, causing intra-testicular haematomas and fibrosis. These complications are much less likely to occur with mTESE compared to cTESE (17–19). This procedure involves making a generous equatorial incision in the tunica albuginea and bivalving of the testis. Under high magnification of the surgical microscope, very small biopsies are removed from areas showing dilated opaque tubules, which are more likely to contain spermatozoa. The bivalving allows the surgeon to reach deeper areas around the hilum and gives access to the entire testicular parenchyma. Thus, mTESE allows for multiple tiny targeted biopsies with sampling of the entire testicular parenchyma as opposed to the random, large and superficial biopsies that cTESE provides. The microsurgical technique also allows visualization of the blood vessels which minimizes vascular injury and allows for accurate haemostasis using microbipolar diathermy should a vascular injury occur (17).

MicroTESE has been reported to show higher success rates and lesser associated testicular damage compared to cTESE in NOA. A meta-analysis of 15 studies with a total of 1,890 men with NOA revealed a 1.5 fold increased chance of success when mTESE was used compared to cTESE (52% SSR rate vs. 35% SSR rate respectively) (20). However a much larger and more recent meta-analysis of 117 studies including 21,404 men with NOA revealed no difference between the two techniques in recovery rate [46 (43; 49)% for cTESE vs. 46 (42; 49)% for mTESE] with an overall SSR rate of 47(45:49)% (21). Interestingly, the SSR rate decreased as the number of men with KS included in the studies increased (*P* < 0.01). Testicular sperm retrieval rates for KS patients with NOA are similar to other men with NOA. A literature review including 13 studies and 373 men with KS demonstrated an overall success rate for SSR of 44% with a range of 16–60% (14) and another review including 19 studies and 668 men with KS revealed a positive SSR rate of 49.6% with a range of 16–69% (15). The success rate was higher for mTESE [55% (61/110)] compared to cTESE [42% (95/228)] (14). On the other hand a more recent systematic review and meta-analysis of 37 studies investigating sperm recovery in 1,248 men with KS using a variety of SSR techniques demonstrated similar retrieval rates for mTESE [45 (38; 52)%] compared to cTESE [43 (35; 50)%] and an overall rate of 44 (39:48)% (22). There was also no difference whether the retrieval was a unilateral or bilateral surgical procedure.

These studies indicate a wide range of SSR rates in both NOA and KS patients. Discrepancies in results can be attributed in part to the differences in data collection and analysis. Many of these studies failed to take into account other variables such as varicocele, cryptorchidism, prior treatment for conditions such as cancer, or hormone replacement therapy etc. that may all affect the success rate of SSR and introduce bias into the data analysis. Furthermore, outcomes were reported for SSR for the different techniques but were not necessarily confined to a single attempt at SSR. These data are also influenced by the inclusion of separate studies that measure SSR rates using either mTESE or cTESE alone, rather than by direct comparison. Interestingly, when Corona et al. (21) restricted their data analysis to eight studies performing direct comparison of the two methods, a significant difference was found overwhelmingly in favor of mTESE (57% SSR rate) over cTESE (39% SSR rate) in NOA patients, confirming previous direct comparison studies (20, 23). In a similar systematic review of KS patients, none of the studies included direct comparison of these two methods (22). Although many of the systematic reviews for SSR in KS men appear to be statistically robust encompassing a large cohort of studies over a relatively long period of time, many of the patient numbers in the individual studies are small, some with only 2 patients, rendering the percentage recovery rate an unreliable measure of outcome in these particular cases. In addition, a proportion of these studies included men who were Klinefelter mosaic, who have significantly higher rates of spermatogenensis and higher success rates with SSR (24).

Differences in SSR success rates may also be explained by differences in patient cohorts across the globe, severity of co-morbidities, and lifestyle factors. The success of SSR is influenced by surgical technique and operative time, as well as the skills of the surgeon and embryology team and the time allocated to searching for spermatozoa in the processed tissue (25, 26). In up to 37% cases, spermatozoa may be found only after 4 h of processing and searching (27). In cases of unsuccessful TESE in KS men, repeat TESE may be considered an option, although to date the number of studies reporting success of rescue TESE in this group of patients is limited. One study showed failure of SSR in a repeat TESE of men with KS where no spermatozoa were found at the first attempt, but the study group only included 2 patients (28), whereas another study of 18 KS men who had a repeat TESE following a previously unsuccessful attempt revealed spermatozoa in 3 cases in a subsequent TESE (29).

## Aneuploidy Rate

The majority of men with KS present with 47, XXY while 10–20% are mosaic Klinefelter's (46 XY/47XXY) or variants with more than one extra sex chromosome (48XXXY, 48XXYY, 49XXXXY) or with partial fragments of supranumery X chromosomes (e.g., 47,X,iXq,Y) (10, 30). KS men with mosaicism are more androgenized than their non-mosaic KS counterparts. A study comparing 86 KS men of whom 6 were mosaic showed, that men with mosaicism were more likely to have more normal hormone levels, higher testicular volumes and fewer co-morbidities (31). Furthermore, this study revealed that 50% men with mosaic KS had sperm in their ejaculates compared to only 7% of non-mosaic KS, hence far less KS men with mosaicism would require SSR. Although success rates for SSR in KS may be as high as 55% as described above, they may be affected by the degree of genetic abnormality. Enatsu et al. (32) managed to obtain very small numbers of spermatozoa from mTESE for a patient with a 47, XXYqs variant of KS, while another case study revealed Sertoli cell only in a patient with a 47,XY,i(X)(q10) karyotype (33). Because genetic variants are rare, most of the studies tend to be case reports, so clearly more studies are needed to fully assess the extent of their impact on spermatogenesis. Although it is possible to retrieve spermatozoa in men with KS, there is considerable debate about the risk of aneuploidy in the offspring. While some studies show a low aneuploidy rate of <7% in spermatozoa from KS men, others show a significant increase in sperm aneuploidy in testicular spermatozoa up to 30% from KS men compared to spermatozoa from normozoospermic or oligozoospermic men with normal karyotypes (9, 34–36). However, numbers in these studies are very small, with a total of only 5 KS men that showed high sperm aneuploidy rates. On the other hand, pregnancy outcome following assisted conception using testicular sperm from non-mosaic KS men shows only a 1% aneuploidy rate in the offspring (37) with pregnancy rates and miscarriage rates being no different to that of other men with NOA (15). These observations have led to the suggestion that euploid spermatozoa can be retrieved from KS men and are likely to have originated from spermatogonia that have lost the supernumery X chromosome during mitosis or from spermatocytes that have lost the X chromosome during meiosis (15). Evidence supporting this comes from investigation of the germinal cells within the testes. Studies have revealed that in testicular foci where spermatozoa are found, a proportion of spermatogonia and all of the spermatocytes were euploid whereas none of these germ cells had a normal chromosomal complement in foci where there is absence of spermatozoa (11, 12, 38, 39). This suggests that development of abnormal sperm cells is arrested at the primary or secondary spermatocyte (or spermatid) stage prior to formation of mature spermatozoa (11).

## Physical Characteristics and Comorbidities

Men with KS present with variable phenotypes, with worsening features seen in those with higher aneuploidy rates. Physical manifestations include decreased facial and body hair, gynaecomastia, decreased muscle tone, tall stature, narrow shoulders and reduced testicular size. In general, reduced testicular size (<12.5 ml) and abnormal testicular histology are associated with worse SSR outcomes (21, 40). Men with KS have lower testicular volume with small firm testes and a volume under 4 ml (41). However, the vast majority of studies have failed to find any correlation between testicular volume and SSR rates in these patients (22, 42–45). The reasons for this are unclear however it may be related to the specific genetic etiology of KS. In this regard, it is of interest that a study comparing SSR rates amongst different etiologies of NOA showed that for men with an average testicular volume of <2 ml, successful SSR outcome was more likely in men with KS than in men without this diagnosis (46). Cryptorchidism which is a leading cause of male infertility, is another co-morbidity with a high prevalence in KS and may affect the outcome of SSR (44). Studies have shown that KS men with a history of undescended testes (UDT) may still have active spermatogenesis provided orchidopexy is performed at an early age. MTESE performed on 29 young adults with non-mosaic KS with a history of UDT had a recovery rate of 31% compared to 38% in non-mosaic KS young adults with ectopic testes (47). Although these recovery rates are encouraging, they were still significantly lower than those from men with NOA with a normal karyotype (66%; *p* < 0.001). Co-morbidities may also include a propensity for insulin resistance, dyslipidemia and obesity as well as an increased risk of thrombosis and cardiovascular disease (48) all of which are associated with impairment of spermatogenesis (49) and may reduce the chances of a successful SSR.

### Endocrine Balance

Ninety % of adults with KS suffer from NOA and hypergonadotropic hypogonadism with low to borderline-normal testosterone levels, increased luteinising hormone (LH), follicle-stimulating hormone (FSH) and oestradiol levels (50). While most studies fail to show an association between testosterone, LH, FSH, inhibin B, AMH and estradiol levels on SSR rates in KS patients (22, 29, 43, 44), the role of testosterone, FSH and LH is not clearly defined. Some argue that higher testosterone and lower LH levels are predictive of successful spermatozoa retrieval (44, 51) while lower testosterone levels are associated with worse SSR outcomes (52, 53). The role of FSH levels as a predictor for successful SSR was found to be variable according to some studies (42, 54). One study found that FSH and LH levels were significantly lower in KS men previously treated with testosterone replacement therapy (TRT) who were unsuccessful with SSR compared to those men where spermatozoa were found (45). It could be argued that serum testosterone levels are not necessarily correlated with intra-testicular levels of this hormone that are essential for spermatogenesis, which may explain why some studies failed to show an association of this hormone with SSR outcome. Indeed many KS patients may already be receiving TRT when they present for infertility investigation due to hypogonadism (48), and there is some debate about the use of TRT in KS patients attending for SSR. A study on ten KS adolescents treated with topical testosterone and aromatase inhibitor therapy for up to 5 years showed a 70% recovery rate for SSR, indicating spermatogenesis was not impaired by this treatment (55). Furthermore, Garolla et al. (45) showed no difference in the success rate of SSR between 111 KS men with or without TRT treatment (33.3 vs. 34.6%, respectively). However, among KS men treated with TRT, those with low FSH and LH levels were unsuccessful with SSR (45). This can be explained by the negative feedback of exogenous testosterone on the hypothalamo-pituitary-gonadal axis which causes a reduction in FSH and intra-testicular testosterone levels, consequently suppressing spermatogenesis. As patient compliance and response to treatment is variable, it may be preferable to stop TRT for 6 months prior to SSR (24). With this in mind, it is recommended that patients who are not hypogonadal should not receive testosterone therapy before SSR due to its potential inhibitory effects on spermatogenesis as discussed above.

**Figure 1:**
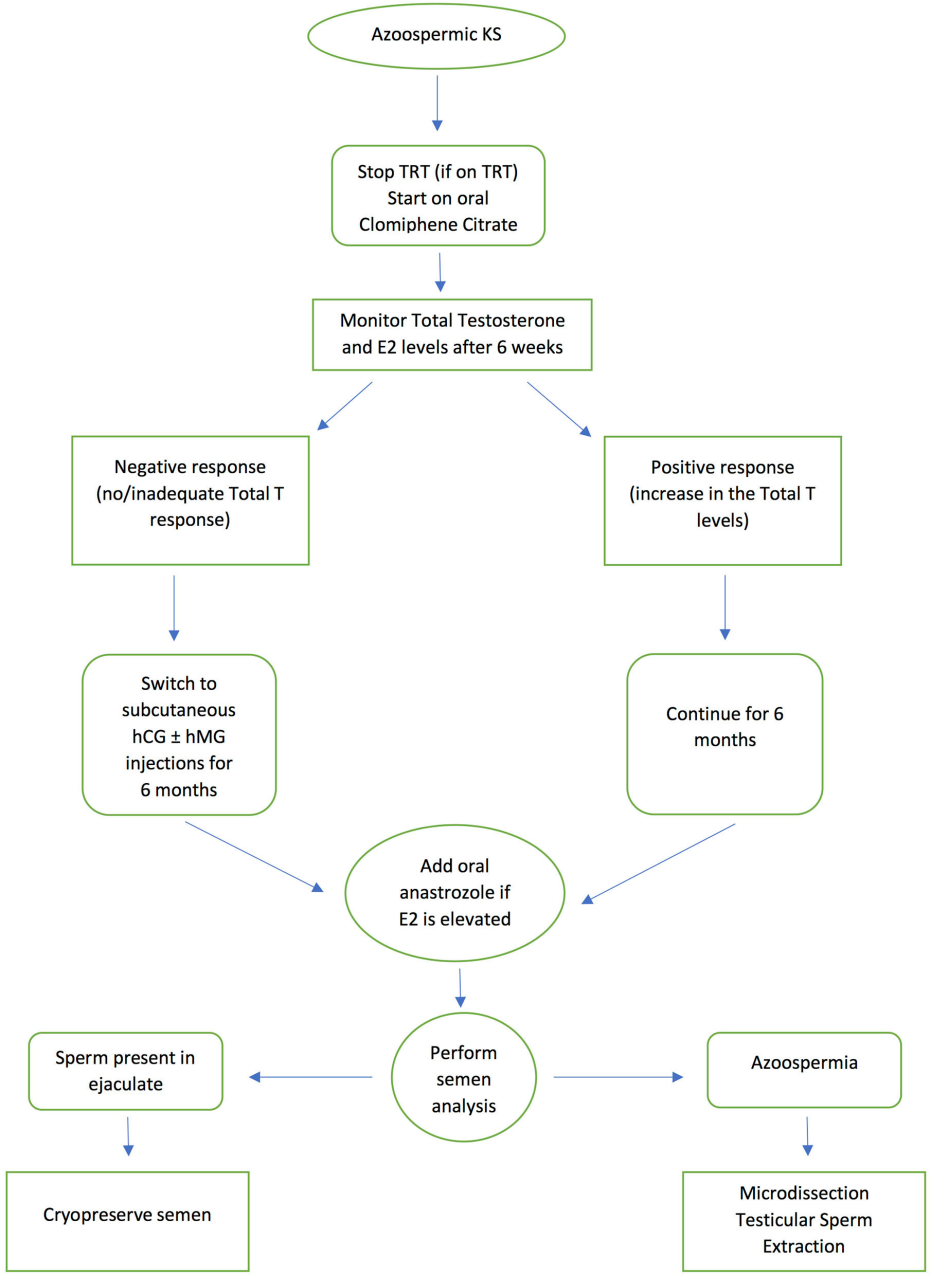
Flow chart of the management of azoospermic KS patients.

Antioestrogens (selective estrogenic receptor modulators -SERMs) such as clomiphene citrate and tamoxifen; human chorionic gonadotropin (hCG) and human menopausal gonadotropin (hMG) have been used successfully in men with NOA prior to SSR (56–59). SERMs act by blocking the negative feedback effect of testosterone on the hypothalamus and pituitary making them insensitive to testosterone, leading to an increase in FSH and LH production, while hCG mimics LH and hMG mimics FSH. Circulating FSH stimulates Sertoli cells while LH will in turn increase intra-testicular testosterone levels. However, this stimulation protocol remains empirical even if the basal hormone levels are within the reference range. Several studies have also shown that empirical hormonal stimulation before SSR in cases of NOA, is associated with a more favorable outcome, with reports suggesting a 15% improvement in men who receive hormonal stimulation before mTESE (56–59). However, there are limited studies on the use of hormone therapy prior to SSR in KS individuals. In one study, SSR was only successful following hormone treatment (60). In this study, the highest retrieval rate was obtained with aromatase inhibitors, probably because aromatase activity, which causes peripheral conversion of testosterone to estradiol, is known to be higher in men with KS. Ramasamy et al. (61) treated KS patients with aromatase inhibitors, clomiphene citrate or hCG prior to SSR. Those who responded to treatment with an increase in testosterone >250 ng/dL showed a significantly higher SSR rate than those who failed to respond. Many clinicians now advocate the use of aromatase inhibitors and selective estrogen receptor modulators prior to SSR (24, 62). The current authors' practice is to use clomiphene citrate as a first line approach as it is inexpensive and can be administered orally, before progressing to subcutaneous injections of hCG and hMG in those that do not respond, as evidenced by failure of an increase in testosterone levels. An aromatase enzyme inhibitor such as anastrozole may be added if there is an increase in estradiol levels above the normal reference range. Ideally KS patients are given hormone therapy for 3–6 months before SSR to promote spermatogenesis. Figure 1 presents a flowchart summarizing the patient management pathway for KS men with azoospermia. This protocol is appropriate for all cases of KS, as soon as they are diagnosed with the condition.

### Age

Men with KS show progressive hyalinisation and fibrosis of the seminiferous tubules over time (63, 64). Thus, it has been suggested that the younger the age at which SSR is performed, the better the chances of finding spermatozoa. While some studies have failed to show an effect of age on SSR rates in KS men (9, 22, 45), others state that age was the only significant factor predicting the success of SSR in KS patients (43, 46, 52, 61, 65, 66). Furthermore, there is a significant reduction in the success rates of SSR in KS above the age of 35 (43, 61, 65, 67). The differences in observations are likely due to the fact that spermatozoa quality in KS is optimal only during a small window of time during adolescence and early adulthood and most SSR procedures are performed on men who are considerably older. Indeed when SSR is performed on a wider age group of people with KS, younger people (15–19 years) are significantly more likely to have a positive SSR outcome than older people (20–61 years) (45 vs. 31% SSR rate, respectively) (44). Consequently, it may be advisable to perform pre-emptive SSR and sperm cryopreservation for young men with KS before they are even planning to start a family and to consider the risks and benefits of SSR in adolescents, especially with regard to ethical considerations.

## Conclusions

Infertility is a characteristic of KS, however with current understanding of the pathogenesis of this syndrome and advances in assisted reproductive techniques, KS men are able to father their own biological children. There is good evidence that KS men with NOA can be successfully treated with SSR to retrieve spermatozoa from localized regions of the seminiferous tubules. As KS men generally have smaller testicular volume, mTESE is therefore advised as the treatment of choice to minimize localized testicular damage. While there is some disagreement in the literature regarding the impact of testicular volume, hormone balance and age of the patient on the success rates of SSR, overall, increasing age appears to be a significant factor. Furthermore, these factors may be indicative of the quantity and quality of spermatozoa retrieved during these procedures rather than the predictability of finding spermatozoa. Stopping testosterone replacement therapy and empirical hormonal stimulation, improves outcomes of both TESE and rescue TESE in KS men. However, more studies are required to standardize protocols and confirm the efficacy of these treatments. The evidence advocates performing SSR in younger men, however as levels of testosterone and subsequent androgenisation may be normal in the majority of KS men (63), the syndrome may remain undetected in many individuals, and often it is not until they experience difficulties in conceiving with their partners later in life that the cause of their infertility is discovered. Ethical concerns have been raised regarding the use of testicular spermatozoa for assisted conception treatment as it is not clearly established whether spermatozoa retrieved by SSR from non-mosaic KS individuals are euploid. Nevertheless, it is encouraging that several studies have reported live births of healthy offspring under these circumstances [reviewed by Corona et al. (22)].

## Author Contributions

AR and SH contributed to the conception and design of the review. YK wrote the manuscript and it was critically revised by SH. AR reviewed all drafts and provided support throughout the writing of the manuscript. All authors approved the final manuscript.

## Conflict of Interest

The authors declare that the research was conducted in the absence of any commercial or financial relationships that could be construed as a potential conflict of interest.
